# Sex and interleukin-6 are prognostic factors for autoimmune toxicity following treatment with anti-CTLA4 blockade

**DOI:** 10.1186/s12967-018-1467-x

**Published:** 2018-04-11

**Authors:** Sara Valpione, Sandro Pasquali, Luca Giovanni Campana, Luisa Piccin, Simone Mocellin, Jacopo Pigozzo, Vanna Chiarion-Sileni

**Affiliations:** 1 Present Address: CRUK Manchester Institute and The Christie NHS Foundation Trust, The University of Manchester, Manchester, M20 4GJ UK; 2 Melanoma and Esophageal Cancer Unit, Istituto Oncologico Veneto-IRCCS, Via Gattamelata 64, 35128 Padua, Italy; 3 Department of Surgery, Oncology and Gastroenterology, University of Padova, 64 Gattamelata St, 35128 Padua, Italy; 4 Surgical Oncology, Veneto Oncology Institute, Via Gattamelata 64, 35128 Padua, Italy; 5 Department of Surgery, Fondazione IRCCS Istituto Nazionale dei Tumori, via G Venezian 1, 20133 Milan, Italy; 6 Department of clinical medicine and surgery, Medical Oncology Unit, University of Naples Federico II, Via S Pansini 5, 80131 Naples, Italy

**Keywords:** Metastatic melanoma, Ipilimumab, Immunotherapy, Toxicity, Interleukin-6, Autoimmunity, Immune-related adverse events

## Abstract

**Background:**

Ipilimumab is a licensed immunotherapy for metastatic melanoma patients and, in the US, as adjuvant treatment for high risk melanoma radically resected. The use of ipilimumab is associated with a typical but unpredictable pattern of side effects. The purpose of this study was to identify clinical features and blood biomarkers capable of predicting ipilimumab related toxicity.

**Methods:**

We performed a prospective study aimed at analyzing potential clinical and biological markers associated with immune-related toxicity in patients treated with ipilimumab (3 mg/kg, q3w). We enrolled 140 consecutive melanoma patients treated with ipilimumab for metastatic disease. The following prospectively collected data were utilized: patient characteristics, previous therapies, level of circulating biomarkers associated with tumour burden or immune-inflammation status (lactic dehydrogenase, C-reactive protein, β2-microglobulin, vascular endothelial growth factor, interleukin-2, interleukin-6, S-100, alkaline phosphatase, transaminases) and blood cells subsets (leukocyte and lymphocyte subpopulations). Logistic regression was used for multivariate analysis of data.

**Results:**

Out of 140 patients, 36 (26%) experienced a severe adverse event, 33 (24%) discontinued treatment for severe toxicity. Among the immune-profile biomarkers analyzed, only interleukin-6 was associated with the risk of toxicity. Female patients had a further increase of immune-related adverse events. Low baseline interleukin-6 serum levels (OR = 2.84, 95% CI 1.34–6.03, *P *= 0.007) and sex female (OR = 1.5, 95% CI 1.06–2.16 *P *= 0.022) and were significant and independent risk factors for immune related adverse events.

**Conclusions:**

Baseline IL6 serum levels and female sex were significantly and independently associated with higher risk of severe toxicity and could be exploited in clinical practice to personalize toxicity surveillance in patients treated with ipilimumab.

**Electronic supplementary material:**

The online version of this article (10.1186/s12967-018-1467-x) contains supplementary material, which is available to authorized users.

## Background

Melanoma is a highly immunogenic tumor, as a consequence of the high somatic mutation rate and expression of neoantigens characterizing this malignancy [1, 2]. However, immunotherapy treatments, such as active specific immunization (i.e., vaccines) and immuno stimulating cytokines (e.g., interferon alpha and interleukin-2) basically failed to significantly improve patient survival [3, 4].

Conversely, elucidation of cellular and molecular mechanisms underlying the suppressive immunological checkpoints has led to meaningful results. Firstly, ipilimumab—a fully humanized monoclonal antibody blocking the co-inhibitory molecule cytotoxic T-lymphocyte antigen 4 (CTLA4)—proved to be effective in patients with metastatic melanoma at the dose of 3 mg/kg q3w. The major effect is the increase of survival, with approximately one in six patients experiencing long-term survival [5].

However, treatment with ipilimumab may be associated with severe autoimmune toxicity, usually according to a specific time pattern. The current toxicity scoring system is derived from the National Cancer Institute’s Common Terminology Criteria for Adverse Events (CTCAE). In clinical trials, patients treated with ipilimumab at the registered dose of 3 mg/kg q21 days for 4 cycles experienced grade (G) 3 (not life-threatening) or G4 (life-threatening) adverse events (AEs) in up to 19.1 and 3.8% of patients, respectively, due to the development of autoimmunity effects. Of note, the reports from “real life” settings (i.e., outside clinical trials) describe even higher toxicity rates, G3–4 AEs being observed in up to 30% of patients [6]. The risk of ipilimumab toxicity is not limited to the treatment course but subsists after therapy completion (late or delayed AE). Apparently, the occurrence of a severe AE does not compromise the activity and efficacy of ipilimumab treatment, but can potentially be fatal and prolong exposure to immunosuppressant therapies (mainly corticosteroids) used to counterbalance the excessive immune upregulation by negative checkpoint inhibitors [7]. Up to date, no risk factors for toxicity have been found. The identification of patients who have a higher likelihood to develop severe AEs could help personalize the safety survey, for example by means of telephonic interviews between visits or biochemical monitoring (e.g., hypophysis hormone levels) after treatment conclusion.

The purpose of the present study was to screen easily accessible biomarkers of ipilimumab toxicity risk, with the final aim to identify tools for personalized safety monitoring. With this purpose, we collected and analyzed the clinical and anthropometric features with potential influence on inflammatory or immunological status (age, sex), and tumor burden surrogate biomarkers (S-100 and lactic dehydrogenase [LDH] [8, 9]). Then, to investigate the inflammatory status of patients, we performed a study of blood biomarkers of inflammation or infection that could easily be used in an outpatient setting, such as proteins and cytokines associated with inflammation, immune reaction or autoimmune disease activity (C-reactive protein [CRP] and beta-2 microglobulin) [10–12], vascular endothelial growth factor-A [VEGF] [13], interleukin 2 [IL2] [14, 15], interleukin 6 [IL6] [16]). In addition, peripheral blood granulocytes and lymphocyte subpopulations were counted to assess the possible influence on toxicity of different leukocyte subpopulations.

## Methods

### Patients and therapy

An observational prospective study was started at the Veneto Institute of Oncology (IOV) in December 2010, the main inclusion criterion being the administration of ipilimumab 3 mg/kg every 3 weeks for metastatic melanoma. Patient characteristics were recorded from clinical records; age and sex were included in the analysis. Then, to investigate the inflammatory status of patients, we performed a study of biological blood markers of inflammation or immune activation, already used as biomarkers in clinical settings, but that had never been investigated in an immunotherapy toxicity context. The biomarker panel to investigate was decided by consensus (based on bibliography data) after discussion among the authors and expert collaborators; the selection was based the potential role of the markers as surrogate biomarkers for melanoma tumour burden or as inflammatory mediators that may influence tumour response as well as autoimmunity occurrence. Blood tests and clinical examination were performed before every ipilimumab cycle (time window from 1 week to the same day before administration) and then according to scheduled follow-up surveillance (first visit 2 weeks after treatment completion and then approximately every 12 weeks). In particular, blood was analyzed, within 2 h from phlebotomy, for melanoma tumour burden surrogates (LDH, S-100 [8, 9]), acute phase proteins and cytokines associated with immune reaction, inflammation or autoimmune disease activity (CRP [10], beta-2 microglobulin [11, 12], VEGF [13], IL2 [14, 15], IL6 [16]). LDH was measured by means of the kinetic method optimized according to the German Society of Clinical Chemistry (Roche Cobas 8000 c720, Cobas Core and ISE 1800), CRP was measured with nephelometric method (Siemens Vista), beta2-microglobulin was measured with immunonephelometric method (Siemens Vista), VEGF was measured with immunoenzymatic method (Thermo Scientific), IL2 was measured with immunoenzymatic method (Thermo Scientific), IL6 was measured with chemoluminescent immunoenzymatic method (Beckman Coulter DXI 800), S-100 was measured with chemoluminescent immunodosing (Diasorin Liaison XL LAS). In addition, to assess the role of different leukocyte subpopulations in treatment efficacy and toxicity, peripheral blood leucocyte (cytometric method; Siemens ADVIA 2120i) and lymphocytes subpopulations (fluorochrome-labeled antibody flow cytometry to identify membrane positivity for CD3, CD4, CD8, CD16, CD19 and CD56; Beckman Coulter FC 500 and Integrated Cytometry Solution) were included into the study. The following auto-antibodies were also searched (indirect immunofluorescence, chemoluminescent immunodosage and immunoenzymatic method) in the plasma of patients: anti-thyroperoxydase (Diasorin Liaison XL LAS), anti-thyroglobuline (Diasorin Liaison XL LAS), anti-neutrophil cytoplasmic and nucleus antigens (Menarini Zenit-up Inova kit and Nikon Eclips 55i Microscope), anti-glutamic acid decarboxylase (anti-GAD) (Grifols Triturus, Euroimmun kit), and anti-adrenal glands (Grifols Triturus, Euroimmun kit). All blood tests were performed, as part of a research biomarker project, in a Good Clinical Practice accredited clinical laboratory (Unità Operativa Complessa Medicina di Laboratorio, Azienda Ospedaliera di Padova laboratory within Istituto Oncologico Veneto) according to automated or semi-automated assay manufacturer standard operating procedures. Only one patient was lost after first follow up visit. In case of toxicity, blood tests and examination took place during an urgent unscheduled visit. Occurrence and outcome of AEs (according to CTCAE v.4.0), date of last follow up and cause of death (melanoma or other) were collected from clinical records. All patients gave informed consent to the treatments and to the use of their clinical records for scientific purposes. The study was performed under local Institutional Review Board approval.

### Statistical analysis

We used logistic regression analysis, corrected for the bias in prediction error estimates [17], to examine the association between toxicity and above mentioned biomarkers. The algorithm was constructed including stratification for time of observation. The model was fitted to data using Wald test with Bonferroni correction for multiple testing to assess the statistical significance of each covariate included in the model. Fast-backward method (with Akaike Information Criterion [AIC] as a stopping rule) was applied to test the covariates in the final model. Stratification for the time of observation was included in the model. Performance of this model was measured with the Receiver Operating Curve, Harrell’s C-Index and standard error derived by the estimation were reported; smooth calibration was evaluated with shrinkage slope (after 200 bootstrap replications). The predictive effect of the model was then validated using bootstrap methodology (200 replications), as advised for small datasets [18]. Visual tree method was used to report cluster analysis for covariates. Overall survival (OS) was calculated from first ipilimumab administration to date of death or last follow-up. OS was estimated with the Kaplan–Meier survival method and log rank test. Two-sided P-values were reported. Statistical analysis was performed with R 3.0.2 (survival, ROCR and rms libraries, R Foundation for Statistical Computing, Vienna, Austria).

## Results

### Patients’ characteristics

The characteristics of the 140 patients included in this study and baseline biomarkers levels are summarized in Table 1; median CD3 positive lymphocyte count, IL2 and S-100 levels were superior to the value of the average healthy population. However, baseline total lymphocytes (rho = − 0.01, *P *= 0.908), CD4 (rho = − 0.09, *P *= 0.497) CD8 (rho = 0.12, *P *= 0.391) or CD3 positive (rho = − 0.03, *P *= 0.798) lymphocyte count did not correlate with IL2 levels.

**Table 1:** Patient characteristics and biomarkers

Patients characteristics	Median or *N* (range or %)
Sex	
Male	86 (61.4)
Female	54 (38.6)
Age	63.0 (27.0–85.0)
Number of previous treatments	1 (0–4)
Follow-up (months)	9.8 (2.4–53.6)

Biomarker (normal range)	Median (range)
White blood cells (4.40–11.00 × 10^6^/L)	6.2 (2.3–17.5)
Eosinophils (0–0.50 × 10^6^/L)	0.08 (0.01–0.89)
Neutrophils (1.80–7.8 × 10^6^/L)	4.0 (1.1–16.2)
Lymphocytes (1.10–4.80 × 10^6^/L)	1.3 (0.7–2.5)
CD3+ (7.0–27.0%)	71.0 (42.0–92.0)
CD4+ (32–52%)	39.0 (17.0–73.0)
CD8+ (16–33%)	23.0 (5.3–79.0)
NK (7.0–27.0%)	18.0 (5.6–35.6)
CD3/CD16/CD56+ (1–11%)	3.0 (1.0–13.0)
LDH (< 1, × UNL)	0.9 (0.4–11.56)
CRP (0–6 mg/L)	6.7 (2.9–214.0)
β2-microglobulin (1.09–2.53 ng/L)	2.3 (1.2–7.2)
IL6 (0–5.9 ng/L)	3.5 (2.0–658.0)
IL2 (0–2 ng/L)	7 (2–28.3)
S-100 (0.00–0.15 μg/L)	0.6 (0.03–97.0)
VEGF (62–707 ng/L)	431.5 (3.4–2100.0)

The table describes the features of the patients included in the study. Of note, most of the biomarkers lied within the normal ranges with the exception of CD3 positive lymphocytes, IL2 and S-100 levels, that were superior to the value of the average healthy population

At data cut-off, 56 patients (40%) were alive after a median follow up of 9.8 months (range 2.4–53.6); median OS was 9.6 months (range 0.8–33.1). One and 2-year survival rates were 39.0% and 20.9%, respectively. Two-thirds of the patients (*N *= 93, 66%) completed the 4 cycles of therapy. Of the remaining patients, 33 (24%) discontinued treatment for toxicity and 14 (10%) developed symptomatic central nervous system (CNS) metastases requiring steroids or rapid performance status (PS) worsening related to disease progression. No patients had previously received anti-PD1 treatments.

### Toxicity and prognostic factors for immune-related toxicity

AEs are reported in Table 2 and reflect the typical toxicity pattern for ipilimumab in a real world setting. Sixty-five of 140 patients (46%) experienced some AEs (any grade, with 124 recorded AEs); of them, 49 had more than one AE, the commonest association being skin toxicity and constitutional symptoms (19 patients). Thirty-six patients (26%) experienced a severe adverse event (2 patients had 2 concomitant G3–4 AE, with a total of 38 recorded G3–4 AEs). Of note, two of them had late events (one G4 diarrhea 3 months after treatment completion and one G3 diarrhea plus hypophysitis 5 months after treatment completion).

**Table 2:** Adverse events

Adverse event	*N* (%), tot = 140 patients	G3–4 *N* (%), tot = 140 patients
Cutaneous	52 (37)	5 (4)
Pruritus	22 (16)	3 (2)
Rash	24 (17)	2 (1)
Vitiligo	6 (4)	0
Gastrointestinal	30 (21)	21 (15)
Diarrhea	21 (15)	19 (14)
Pancreatitis or lipase/amylase increase	5 (4)	2 (1)
Nausea/vomit	3 (2)	0
Constipation	1 (1)	0
Constitutional symptoms	21 (15)	0
Fatigue	13 (9)	0
Fever	7 (5)	0
Headache	1 (1)	0
Endocrine disorders	12 (9)	11 (8)
Hypophysitis	10 (7)	10 (7)
Thyroiditis	1 (1)	0
Hyperglycemia	1 (1)	1 (1)
Other	9 (6)	1 (1)
Arthralgia	5 (4)	1 (1)
Hepatotoxicity	2 (1)	0
Anemia	1 (1)	0
Posterior uveitis	1 (1)	0

The most frequent adverse events by all grades were cutaneous toxicity. On the other hand, gastrointestinal events accounted for the majority of severe (G3–4 according to the common terminology criteria for adverse events) toxicities. Patients may have more than one toxicity event, in particular, out of 140 patients, 65 (46%) experienced some AEs and of them, 49 had more than one AE, for a total of 124 total recorded adverse events

Grade 3–4 diarrhea, which occurred in 19 patients (14%), was the most frequent cause of treatment discontinuation due to toxicity, followed by hypophysitis, which occurred in 9 patients (6%). Patients experiencing G3–4 AEs remained on corticosteroid therapy for a minimum of 4 weeks, to a maximum of 8 months of mineral-corticoid replacement in a case of hypophysitis (treatment ongoing). Investigated serum antibody titers did not correlate with occurrence of AEs. Of note, we did not observe any correlation between baseline anti-thyroperoxydase titer and the occurrence of thyroiditis. One patient developed anti-GAD antibodies after treatment completion, without evidence of any AEs. One death was suspected to be caused by refractory hypophysitis because of clinical presentation with asthenia and declining PS associated with low Adrenocorticotropic Hormone and ionic imbalance, which worsened despite corticosteroids; however, the autopsy found evidence of immune aggression neither in the hypophysis nor in other organs.

**Fig. 1:**
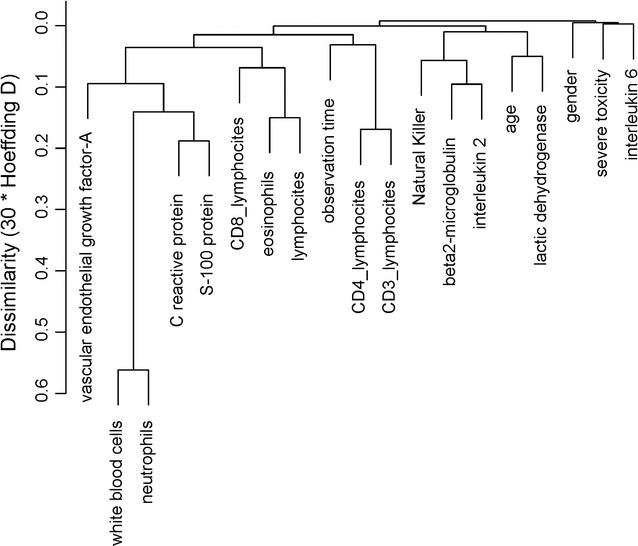
Hierarchical cluster analysis of covariates. The aggregation of covariates is represented in the cluster analysis using Hoeffding’s D as the (dis)similarity measure. This graphically presents the information concerning which observations are grouped together at various levels of similarity and dissimilarity. Vertical lines extend up for each observation, and at various (dis)similarity values, these lines are connected to the lines from other observations with a horizontal line. The height of the vertical lines and the range of the (dis)similarity axis give visual clues about the strength of the clustering. The variable severe toxicity is shown in the right upper corner, in proximity with gender and IL6. Toxicity does not cluster with any other investigated markers

The aggregation of covariates is represented in the cluster analysis of Fig. 1. Sex and IL6 aggregate with the variable toxicity, which does not cluster with other investigated factors.

The association between collected clinical parameters, biomarkers and G3–4 AEs was investigated accounting for patient survival. Female patients and those with lower IL6 baseline serum levels had higher risk of developing G3–4 toxicity (OR = 1.5, 95% CI 1.06–2.16 and OR = 2.84 for 1 ng/L variation, 95% CI 1.34–6.03, respectively); at parity of IL6 levels, female patients had a higher risk of toxicity. These two variables were also the only significant after backward selection (AIC rule satisfied, Chi square 5.24, *P *= 0.022 and Chi square 7.37, *P *= 0.007, respectively).

**Fig. 2:**
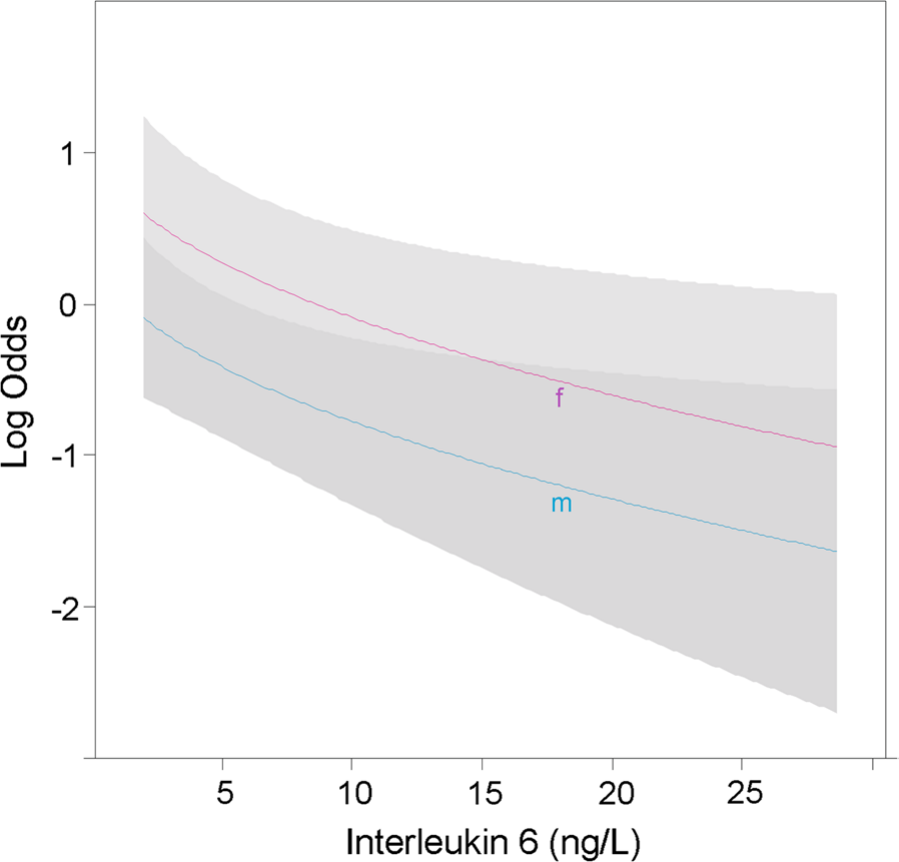
Visual representation of the correlation between baseline IL6 levels, sex and risk of toxicity. The figure depicts the correlation between baseline interleukin-6 (IL6) levels, sex and risk of toxicity (logarithmic scale for the risk). The risk of toxicity decreases with higher IL6 concentrations for both genders. Female patients have, for the same blood concentrations of IL6, a higher risk for toxicity than men

No significant correlation with the subtype of AE emerged from the cytokine analysis, as well as from the analysis of all considered biomarkers (not shown), and we did not find a pattern of acute inflammation biomarker changes at the onset of toxicity compared to baseline (not shown). Correlations and significance level for the full marker panel are reported in Table 3. The cut-off value of 2.5 ng/L (independently of sex) for IL6 provided the best combination of sensitivity of (70.4%) and specificity (66.1%) to discriminate patients with an increased risk of AEs. Figure 2 depicts the correlation between baseline IL6 levels, sex and risk of toxicity.

**Table 3:** Biomarker associated risk of toxicity

Clinical or biological marker	Odds ratio	*P*	95% CI
Interleukin 6	2.84	0.007	1.34–6.03
Sex: female	1.5	0.022	1.06–2.16
Lactic dehydrogenase	1.18	0.645	0.58–2.41
Age	2.82	0.283	0.42–18.81
Interleukin 2	0.74	0.934	0.00–1025.23
Beta2-microglobulin	0.16	0.164	0.01–1.6
Natural Killer cells	0.63	0.593	0.12–3.67
Total lymphocytes	0.28	0.314	0.02–3.36
CD3 lymphocytes	0.41	0.841	0–2500.35
CD4 lymphocytes	2.93	0.722	0.01–1096.90
CD8 lymphocytes	14.04	0.461	0.01–15,879.76
Eosinophils	3.28	0.151	0.65–16.63
S-100 protein	1.05	0.489	0.91–1.21
C reactive protein	2.08	0.308	0.51–8.52
White blood cells	15.02	0.303	0.09–2621.67
Neutrophils	0.59	0.704	0.04–8.95
Vascular endothelial growth factor-A	0.65	0.748	0.04–9.30

The table resumes the odds ratios (OR) for ipilimumab toxicity and significance levels for the markers analyzed in the study (multivariate analysis). Only interleukin 6 and sex had a significant association with the risk of immune-related adverse events (independently of toxicity subgroup). OR for continuous variables refers to the cumulative OR for one unit increase

**Fig. 3:**
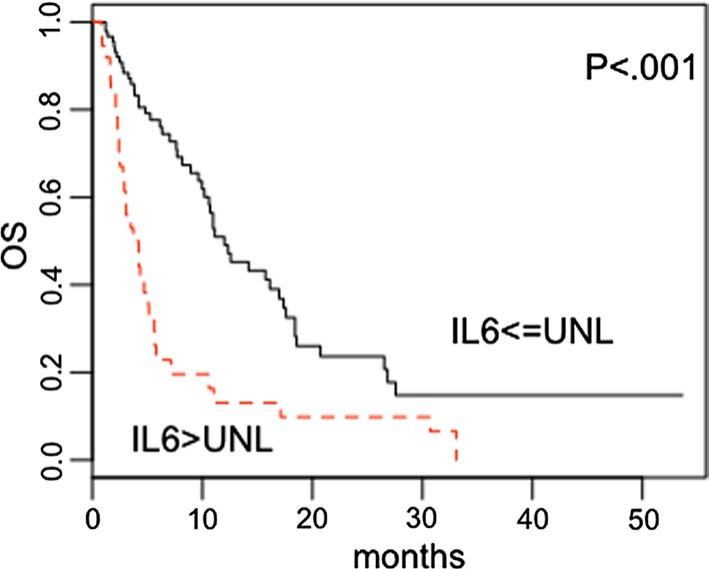
Survival of patients according to IL6 levels. Kaplan–Meier curves showing overall survival (OS) of patients relative to baseline circulating IL6 concentration. The continuous line presents patients with normal baseline IL6, the dotted line the patients with elevated baseline IL6. *UNL* upper normal limit

These findings were validated using bootstrap analysis (Additional file 1: Figure S1), and the calibration curve after bootstrap is showed in Additional file 2: Figure S2; C-index was 0.65, standard error was 0.038. Remarkably, as shown in Fig. 3, patients with normal baseline circulating IL6 had a longer survival compared to patients with elevated IL6 (median survival 12 and 3.37 months, respectively; P < 0.001). We found no significant difference of outcome for patients who had severe AEs compared to patients who had not (not shown).

Nine patients experiencing G2–4 AEs were treated with an anti-PD1 antibody (pembrolizumab or nivolumab) upon disease progression; none of them had severe AEs with anti-PD1 therapy (one patient who had previously suffered from G3 arthritis experienced G1 arthritis after three pembrolizumab courses, resolved with short term low-dose corticosteroid therapy; one patient who previously had G2 pruritus developed transient and self-limiting G1 pruritus after the first course of pembrolizumab). Of note, two patients who had interrupted ipilimumab treatment because of G3 diarrhea and one patient who had interrupted ipilimumab because of G3 hyperglycemia were treated with anti-PD1 s and did not experience any AEs, after a treatment time span between 2 and 8 months.

## Discussion

In the era of immunotherapy, clinicians need tools to personalize immune-toxicity surveillance in order to implement patient safety and resource management [19]. The management of autoimmune toxicity in patients receiving immunotherapy, such as ipilimumab and may be challenging, requires experienced multidisciplinary teams; remarkably, despite appropriate patient education and guidelines for AE treatment, fatal events have occurred in most studies and case series. Moreover, AE management represents an important economic burden [20] which adds to the already high drug costs. It is intuitive to hypothesize that early discovery of AEs by means of personalized monitoring could allow prompt treatment, thus reducing the risk of severe complications, prolonged immunosuppression and associated comorbidities and costs. Strikingly, AEs may occur after completion of treatment with ipilimumab, thus making patient follow-up a challenging task. Taking these considerations together, identification of patients at risk of developing severe AEs is of paramount importance to plan personalized surveillance.

With the advent of second generation anti-immune checkpoint monoclonal antibodies, ipilimumab is no more the standard first line immunotherapy for metastatic melanoma patients; however, it is approved in the adjuvant setting and is still a second line option for patients progressing after anti-PD1 inhibitors and is administered as first line treatment in combination with nivolumab. As consequence, although the role of the biomarkers that we investigated is unknown in contexts of combination immunotherapy and anti-PD1 drugs, the present study could have a clinical relevance in a large patient population and, to our knowledge, is one of the first planned to look for predictors of immune-toxicity [21, 22].

Among a wide range of biomarkers considered, baseline levels of IL6, a well-known pro-inflammatory cytokine, can stratify the risk of developing AEs: in particular, the lower the level of blood IL6, the higher is the risk of AEs. Moreover, female patients have an increased risk than males with the same IL6 concentration. This may have implications for establishing personalized follow-up strategies for these patients, for example by means of intensified monitoring between standard appointments when an elevated risk for AEs is present. This would identify autoimmune toxicity at onset and, as a consequence, possibly improve patient safety and reduce Health System expenses associated to severe and long-established autoimmune toxicity treatment and comorbidities.

CTLA4 blockade by ipilimumab provides suppression of the inhibitory signal to T-cells and increases the chances for activation against tumour cells. Activation of effector T-cells by CTLA4 is not antigen-specific, and the details of the process of tumour clearance and aggression of bystander cells are not completely understood. In fact, the pattern of immune deregulation occurring in individuals or animals with CTLA4 constitutive impairment does not completely match with the most frequent AEs described for anti-CTLA4 antibodies, thus suggesting a toxicity mechanism for these drugs that is not limited to CTLA4 inhibition [23, 24]. In this scenario, the inflammatory environment could play a pivotal role in regulating the development of an autoimmune disease.

Looking for a possible association between treatment response and toxicity is challenging for at least two reasons. Firstly, although immunosuppressive therapy administered to manage AEs is considered not detrimental for anti-tumour response, its real impact on anti-tumour immune activation is unknown. Indeed, we could argue that patients who have ipilimumab-induced AEs might have a better clinical effect than patients without AEs, but this is offset by the steroids necessary to resolve the toxicity, with the final result of making the patients no more responsive than others. Secondly, the probability of experiencing delayed AEs depends on patients’ survival. Intuitively, a patient who dies because of rapid melanoma progression will not have any possibility to develop late AEs, despite an environment potentially favoring autoimmunity. However, the study performed on all the patients of the Ipilimumab Italian Expanded Access Program found no association between effectiveness and occurrence of any AEs [25], this finding being confirmed in our cohort. In contrast, there appears to be a correlation between severe AEs and outcome for melanoma patients treated with anti-PD1s [26], which calls for further investigation in this field.

IL6 is an acute phase cytokine usually secreted during infections or tissue damage and its production is rapidly switched off after healing [27], but an aberrant production has been associated with several aspects of cancer biology [28].

In our cohort, patients with higher levels of IL6 have lower risk of AEs; conversely, lower baseline levels of IL6 are associated with higher risk of AEs. Remarkably, we found that metastatic melanoma patients with normal IL6 serum levels had longer survival after ipilimumab treatment, although IL6 baseline concentration didn’t retain it’s significance when we analyzed its prognostic value for survival in a multivariate analysis that also identified baseline LDH and neutrophil count as prognostic biomarkers [29], while LDH and neutrophil count did. These results are consistent with the hypothesis that IL6 both increases tumour invasiveness and polarizes inflammation towards immune-suppression [30–36]. In this context, metastatic melanoma may induce chronic high level of IL6, which can both confer aggressiveness and compromise the immune-inflammatory regulation, impairing the immune response elicited by CTLA4 blockade. Conversely, patients with low, normal physiological levels of IL6 (the cut-off we found is within the normal range) have more probability to respond to ipilimumab, but their immune system will also be at risk of significant AEs.

Given that females are at higher risk of several autoimmune diseases, it is not surprising that females have a greater risk of AEs than males with the same levels of IL6 and time of observation. The results of our study support the hypothesis of a significant role for sex-specific factors, for example hormones, in immune-modulation. Interestingly, no sex effect was observed in immunotherapy prognostic studies; nonetheless, the prognosis of primary melanoma is different for the two sexs, in favor of female patients, and the impact of the endocrine system on immune regulation in patients with melanoma is yet to be explored [37].

Consistently with the hypothesis of a mainly cytotoxic lymphocyte mediated toxicity [38], we identified no correlation between auto-antibodies and AEs. Interestingly, we found CD3 positive lymphocyte count, IL2 and S-100 values superior to the average healthy population range and, although increased S-100 concentration can be likely explained by the metastatic burden, the observation relative to CD3 lymphocytes and IL2 could be associated with the systemic immune stress induced by melanoma and is hypothesis generating.

This study was designed to investigate biomarkers commonly available at clinical laboratories in order to offer easy-to-obtain and reproducible biomarkers of toxicity and did not analyze immunosuppressive blood cells. However, Martens et al. [22] found no association between immunosuppressive blood cells and adverse event occurrence in metastatic melanoma patients treated with ipilimumab.

A subgroup of patients who underwent treatment with anti-PD1 antibodies after severe toxicity from ipilimumab did not experience significant reactivation of AEs, with no evidence of cross-linking autoimmune toxicity, as previously suggested [39]. Of note, the diffusion of checkpoint inhibitors based immunotherapy for a growing number of cancers [40], the use of combination checkpoint inhibitors like ipilimumab plus nivolumab (with a significant risk of immune toxicity) [41–43], coupled with the implementation of ipilimumab for the adjuvant therapy of melanoma [44, 45], should invite to extend the use of sex and IL6 for AEs risk estimation in patients affected with tumours other than melanoma and treatment setting other than metastatic.

In some measure, the calibration and the validation of the statistical model suffered from the small sample size, thus encouraging further validation of our results in larger series that could have a more significant predictive value. Similarly, ad hoc studies are required to assess the importance of IL6 and sex as prognostic factors for immune-mediated toxicity in the context of other immunotherapy regimens.

## Conclusions

In conclusion, although our findings should be verified in other prospective studies, baseline blood IL6 and sex are promising biomarkers for immune-mediated toxicity and could be evaluated before ipilimumab treatment to identify patients at risk of AEs, with the purpose to personalize monitoring during and after the treatment and improve patient safety and resource management. In particular, females with low IL6 baseline serum levels should be carefully monitored for toxicity, including late AEs. These results have implications for patients counseling and for planning appropriate toxicity surveillance even after treatment conclusion.

## Additional files

**Additional file 1.** Model validation. The scatter plot represents the validation of the predicted risk of toxicity model built with bootstrap analysis with gender and interleukin-6 baseline blood concentrations. However limited by the small sample, the validation result is satisfactory; an ideal line at 45° that would represent a perfect match between observations and predictions, and the validation scatter plot has a very limited dispersion.

**Additional file 2.** Model calibration curve. Estimate of calibration accuracy was performed using adaptive spline regression with bootstrap methodology. The line adjacent the ideal line corresponds to the apparent predictive accuracy. The blue line corresponds to corrected estimates.
